# Incident rheumatoid arthritis and work loss: a nationwide sibling comparison study

**DOI:** 10.1093/rheumatology/keag124

**Published:** 2026-03-19

**Authors:** Heather Miller, Gustaf Bruze, Kari Johansson, Johan Askling, Martin Neovius

**Affiliations:** Clinical Epidemiology Division, Department of Medicine Solna, Karolinska Institutet, Stockholm, Sweden; Clinical Epidemiology Division, Department of Medicine Solna, Karolinska Institutet, Stockholm, Sweden; Clinical Epidemiology Division, Department of Medicine Solna, Karolinska Institutet, Stockholm, Sweden; Clinical Epidemiology Division, Department of Medicine Solna, Karolinska Institutet, Stockholm, Sweden; Rheumatology, Theme Inflammation and Ageing, Karolinska University Hospital, Stockholm, Sweden; Clinical Epidemiology Division, Department of Medicine Solna, Karolinska Institutet, Stockholm, Sweden; Department of Public Health Sciences, Thompson School of Social Work & Public Health, University of Hawai‘i at Mānoa, Honolulu, HI, United States

**Keywords:** work loss, rheumatoid arthritis, disability pension, sick leave

## Abstract

**Objectives:**

Rheumatoid arthritis (RA) has been associated with substantial work loss, but advances in treatment have improved clinical outcomes for patients over time calling for an updated assessment. We therefore aimed to examine calendar trends in the association between RA diagnosis and work loss.

**Methods:**

This study included 3850 patients with RA, diagnosed between 2006 and 2020, aged 30–60 years, and their 4422 same-sex siblings. Data on work loss (the sum of sick leave and disability days) were retrieved from the Swedish Social Insurance Agency, from 3 years before up to 10 years after diagnosis. Analyses were stratified by diagnosis period (2006–2011 *vs* 2012–2020).

**Results:**

Starting 13 months before RA diagnosis and peaking in the year thereafter, patients with RA experienced more work loss than their same-sex siblings. This excess was smaller during 2012–2020 than 2006–2011 (26 *vs* 47 days annually, *P* < 0.001). Patients diagnosed in 2012–2020 also had faster declines in work loss 2–10 years post-diagnosis than those diagnosed 2006–2012, with a smaller difference *vs* same-sex siblings (11 *vs* 29 days annually; *P* = 0.001). Work loss was highly skewed, with a small proportion of patients contributing most of the work loss days.

**Conclusion:**

Compared with their same-sex siblings as a surrogate for the counterfactual ideal, RA is still associated with considerable work loss. This difference has, however, declined in recent years, possibly due to earlier diagnosis and improved treatment.

Rheumatology key messages

- Patients with rheumatoid arthritis experience more work loss than their same-sex siblings.
- Work loss in rheumatoid arthritis rises before diagnosis and peaks shortly after diagnosis.
- Patients with rheumatoid arthritis diagnosed recently have lower peak work loss and smaller differences versus siblings.

## Introduction

Rheumatoid arthritis (RA) is a chronic inflammatory joint disease with significant socioeconomic consequences for the individual and society, particularly through increased sick leave and disability. Since the 1980s, studies have consistently shown that individuals with RA experience elevated levels of work loss, including short-term absenteeism and long-term disability [1–5]. More recent research underscores the relationship between disease activity and work loss, with higher disease activity correlated with greater absenteeism [6, 7]. Treatment with disease-modifying antirheumatic drugs (DMARDs) has been associated with lower absenteeism, highlighting the critical role of effective disease management [8, 9].

We and others from Sweden have previously demonstrated that work loss among patients with RA begins to increase 6–12 months before RA diagnosis [5, 10], and remains elevated after diagnosis compared with the age-sex-matched general population [8, 11–13]. Other studies, also reflecting RA care in the beginning of this century, have reported that a third of RA patients become permanently work-disabled within 3 years after disease onset [14]. Advances in RA treatment, including the introduction of biologic and targeted synthetic disease modifying anti-rheumatic drugs (DMARDs) and the implementation of treat-to-target strategies, have improved clinical outcomes over the past decades [15]. How these changes to treatment collectively influence work loss remains uncertain. We have recently reported a 5% reduction in earnings for patients diagnosed between 2006 and 2010 compared with their same-sex siblings over 5 years of follow-up, while no statistically significant impact on earnings was observed for patients diagnosed between 2011 and 2017 [16].

Evolving treatment strategies and efforts to shorten the time from symptom onset to RA diagnosis likely influence the amount and duration of work loss through improved disease control, but knowledge gaps remain regarding how work loss trends vary by diagnosis period. There is evidence of declining work loss, as seen in Finland in the early 2000s, where rates of permanent work disability in early RA decreased [17]. Earlier research has often relied on general population comparators, which are informative but do not differentiate between effects related to RA and effects related to RA risk factors driven by shared genetics and environmental influences such as smoking. To this end, comparing patients to their same-sex siblings would represent a superior means of estimating the effects of RA relative to the counterfactual ideal in terms of patients’ ‘expected’ trajectories of work ability.

The aim of this study was therefore to examine longitudinal trends in work loss as measured by sick leave and disability pension among patients with incident RA compared with their same-sex siblings, by calendar period of RA diagnosis (2006–2011 *vs* 2012–2020).

## Methods

Using Swedish national registers, we compared patients with RA to their same-sex siblings to quantify changes in work loss before and after RA diagnosis in the patient. We used the unique Swedish personal identity number to link data from multiple registers. Ethical approval was granted by the Swedish Ethical Review Authority (DNR 2023-03804-1), with an exemption from informed consent. Patient representatives were not involved in the design or interpretation of this study. No *post hoc* changes were made to the analysis plan, except for adjustments in response to comments from reviewers.

### Participants

We identified patients 30–60 years of age at diagnosis of RA between 2006 and 2020 in the Swedish National Patient Register using International Classification of Diseases 10th Revision (ICD-10-codes) (Supplementary Table S1). This age range typically represents individuals in the prime of their working lives, with higher income levels and greater financial responsibilities. During the study period (until 2020), the earliest age at which an individual could start receiving a public pension in Sweden was 61 years [18].

A diagnosis of RA was assumed if a patient had all of the following: an outpatient RA diagnosis from specialty care, a second RA diagnosis within one year (inpatient or outpatient specialty care) and at least one visit to a rheumatology or internal medicine department listing RA. This definition has been shown to be robust in previous studies using the same data sources [19]. Patients who used DMARDs >6 months before their first RA diagnosis were excluded (to prevent inclusion of prevalent patients with RA). The first date of diagnosis was considered the date of study inclusion (index date) for RA.

### Comparator group

For inclusion in our study, patients with RA were required to have at least one same-sexed full sibling between 30 and 60 years of age at the index date, not >5 years apart in age to minimize age-related differences in work loss patterns while ensuring a sufficient sample size (flowchart in Supplementary Fig. S1). Same-sex siblings were identified in the Multigeneration Register, were required not to have a diagnosis of RA prior to the index date, and were censored if they were diagnosed with RA after the index date.

To describe potential differences between patients with RA and their same-sex siblings, we retrieved data on annual taxable earnings from the Longitudinal Integrated Database for Health Insurance and Labor Market Studies (LISA), and data on educational attainment from the Swedish Education Register. We also retrieved data on comorbidities from the Swedish National Patient Register, including inpatient and non-primary outpatient care, from 5 to 1 years before RA diagnosis.

### Outcome

The Swedish welfare system provides compensation for sick leave and disability pension, which may be complete or partial. The primary outcome was the sum of net sick leave and disability days, collectively described as work loss days. These data were retrieved from the Swedish Social Insurance Agency from 1 January 2003 to 31 January 2021. Work loss serves as an indicator of disease burden, reflecting the physical limitations and economic consequences of RA.

Sick leave was reimbursed by the employer from day 2 to day 14, and episodes longer than 14 days were recorded by the Social Insurance Agency which reimburses the employee due to lost income from day 15 and onwards. We assessed the net number of compensated sick leave days from the Social Insurance Agency, and therefore, episodes of sick leave shorter than 2 weeks paid by the employer were not included.

An individual with a 25% reduced work ability or more (as evaluated by a physician), expected to last for at least 1 year, could receive disability pension. Disability pension (sickness compensation ‘sjukersättning’, age 30–64 years, and activity compensation ‘aktivitetsersättning’, age 19–29 years) was granted in proportion to the reduction in work ability. We retrieved compensation dates for disability pension from the Social Insurance Agency’s database, MiDAS, and total net days per month were calculated by multiplying the number of days by the proportion of compensation received. For example, 2 days with 50% compensation were counted as one full day.

All Swedish residents aged 16 years or older could be granted economic compensation from the Social Insurance Agency in case of sickness, disability or injury. The compensation could take the form of sick leave or disability pension, and it was possible to have both compensations simultaneously, though never exceeding 100% together.

### Follow-up

To analyse the change in work loss from before to after RA diagnosis, we collected data on work loss from 3 years before to up to 10 years after year of diagnosis. Days were collected as annual totals. Monthly work loss totals were also used to provide a more detailed analysis of the relationship between RA diagnosis and work loss, specifically, to investigate when work loss started to rise for patients with RA (relative to same-sex siblings) and when work loss reached its peak.

Participants were followed for a maximum of 10 years after the index date, until the end of the study period (31 January 2021). Participants were censored due to death (RA 3.0%; same-sex siblings 2.0%), emigration (RA 0.4%; same-sex siblings 0.7%) or an incident RA diagnosis (same-sex siblings 1.6%), whichever came first.

### Statistical analyses

Prevalences, means with standard deviations and medians with interquartile ranges were used to describe baseline variables.

The main analysis included stratification by the median year of RA diagnosis (2006–2011 *vs* 2012–2020) to investigate calendar trends. Annual mean differences in work loss between patients and their same-sex siblings were calculated using multiple regression with covariates age, age squared, a dummy variable for the calendar year and same-sex sibling fixed effects to account for shared familial factors. Including age squared accounts for potential non-linear associations between age and work loss. Standard errors were clustered at the same-sex sibling level to adjust for the correlation of observations within same-sex siblings over time. In sensitivity analysis, we included comorbidities at RA diagnosis among the covariates (cardiovascular disease, psychiatric disorder and substance use disorder).

#### Work loss distribution

To examine the distribution of work loss, we categorized patients and same-sex siblings into four groups based on monthly (30-day) work loss: (i) no work loss (0 days); (ii) >0 and ≤15 days; (iii) >15 and <30 days; and (iv) full work loss (30 days). We plotted the distribution of individuals across these categories to capture the variability in individuals’ work loss beyond mean group differences. We also calculated the share of patients with RA and same-sex siblings who were free of registered work loss during three consecutive years around diagnosis (the 1 year before and the first 2 years after diagnosis) by calendar period of diagnosis.

#### Subgroup analyses

Subgroup analyses were conducted to assess potential effect modification by sex, age (split by the median: 30–49 *vs* 50–60 years), low education (a high school degree or less) *vs* high education (some tertiary education or more) and seropositive (ICD10 M05, M06.0L, M06.8L) *vs* seronegative RA. Differences among subgroups were assessed using an interaction term for RA diagnosis and subgroup membership, in the entire cohort (sex, age, seropositivity) or in same-sex siblings where patients and same-sex siblings had the same (low or high) level of education (a restricted study population). In exploratory analyses, we examined work loss for patients with RA and their same-sex siblings in four periods of diagnosis spanning 3 years (2006–2008, 2009–2011, 2012–2014 and 2015–2017). Subgroup analyses were planned beforehand or suggested by reviewers to investigate potential differences by sex, age, educational level and seropositivity, as these are well-known factors influencing disease characteristics and work loss [19–21].

Missing data were minimal (<1% for work loss) and handled through complete-case analysis. Analyses were conducted using Stata version 14.2 (StataCorp, College Station, TX, USA).

## Results

### Participant characteristics

We identified 15 199 patients in the National Patient Register who were diagnosed with RA from 2006 up until 2020, at an age between 30 and 60 years. In this group of patients with RA, 4801 did not have a full sibling, and 6548 did not have a full same-sex sibling who was residing in Sweden at the start of follow-up, who met the age requirements and who was not diagnosed with RA prior to the start of follow-up. The full set of selection criteria resulted in a final study population with 3850 individuals diagnosed with RA and their 4422 same-sex sibling comparators (Supplementary Fig. S1). The median age at diagnosis was 50 years for patients and same-sex siblings in the early (2006–2011) and late (2012–2020) diagnosis periods, 71% were women in both periods and the educational attainment of patients with RA *vs* their same-sex siblings was similar across both diagnosis periods (Table 1, Supplementary Table S2).

**Table 1:** Baseline sociodemographic and clinical characteristics of patients with rheumatoid arthritis and their same-sex siblings according to diagnosis period (2006–2011 and 2012–2020).

	Diagnosis 2006–2011		Diagnosis 2012–2020	
	Patients with RA (n = 1667)	Same-sex siblings (n = 1943)	Patients with RA (n = 2183)	Same-sex siblings (n = 2479)
Women, n (%)	1185 (71.1)	1370 (70.5)	1534 (70.3)	1743 (70.3)
Married, n (%)	825 (49.5)	915 (47.1)	1018 (46.6)	1166 (47.0)
Seropositive RA, n (%)	1118 (67.1)	n.a.	1434 (65.7)	n.a.
**Age (years) at diagnosis/Index date**				
Mean (SD)	48.2 (7.8)	48.3 (7.6)	48.2 (7.8)	48.3 (7.7)
Median (p25–p75)	49.8 (42.3–54.8)	49.7 (42.6–54.5)	49.8 (42.7–54.6)	49.8 (42.8–54.6)
Year of diagnosis, median (p25–p75)	2009 (2007–2010)	2009 (2007–2010)	2016 (2014–2018)	2016 (2014–2018)
**Education and earnings**				
Primary school, n (%)	280 (16.8)	313 (16.1)	247 (11.3)	281 (11.3)
High school, n (%)	888 (53.3)	1022 (52.6)	1149 (52.6)	1291 (52.1)
University, n (%)	498 (29.9)	605 (31.1)	785 (36.0)	901 (36.3)
*Education missing, n (%)*	*1 (0.1)*	*3 (0.2)*	*2 (0.1)*	*6 (0.2)*
Earnings, mean (SD)	240 900 (175 700)	261 500 (181 100)	301 600 (220 800)	317 600 (225 700)
**Comorbidities** ^a^				
Cardiovascular disease, n (%)	128 (7.7)	162 (8.3)	189 (8.7)	198 (8.0)
Psychiatric disorder, n (%)	118 (7.1)	150 (7.7)	196 (9.0)	216 (8.7)
Substance use disorder, n (%)	39 (2.3)	36 (1.9)	46 (2.1)	41 (1.7)
Musculoskeletal disorder (any), n (%)	489 (29.3)	299 (15.4)	726 (33.3)	477 (19.2)
Musculoskeletal disorder (inpatient), n (%)	80 (4.8)	55 (2.8)	95 (4.4)	73 (2.9)

a Retrieved from the National Patient Register, including inpatient and non-primary outpatient care, from 5 years before to 1 year before RA diagnosis.

### Before diagnosis

**Figure 1:**
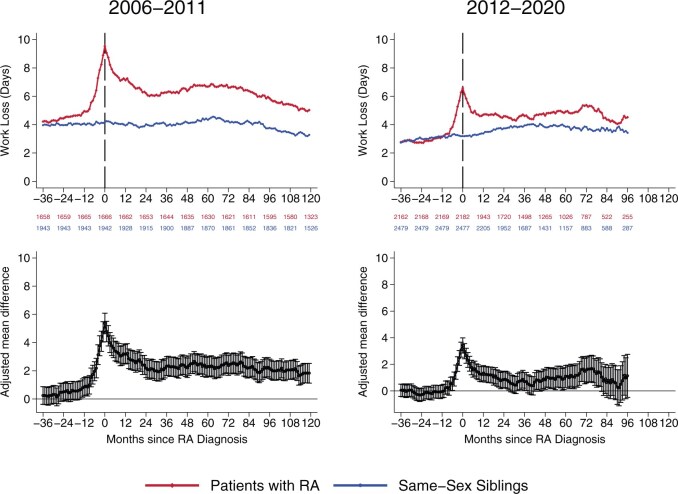
Monthly work loss for patients with RA and same-sex siblings by diagnosis period. Top panels: Mean work loss days by month. Numbers below the *x*-axis indicate the number of individuals with available follow-up data at each time point. Bottom panels: Annual mean differences and 95th percentiles in work loss between patients and same-sex siblings estimated using multiple regression adjusted for age, age squared, and calendar year, with sibling fixed effects. Standard errors were clustered at the sibling level

At 3 years (36 months) before the RA diagnosis, work loss was similar between patients and same-sex siblings in both diagnosis periods (Fig. 1). Comparing 2006–2011 and 2012–2020, patients and same-sex siblings had higher work loss in the earlier than the later period, but 36 months before diagnosis there were no differences between them. For patients diagnosed between 2006 and 2011, work loss began to increase relative to same-sex siblings 19 months before diagnosis [4.6 work loss days in patients with RA *vs* 4.0 days in same-sex siblings, corresponding to an adjusted mean monthly difference of 0.70 (95%CI 0.09–1.32) days]. In contrast, among patients diagnosed between 2012 and 2020, the increase in work loss relative to same-sex siblings appeared later in relation to diagnosis, starting 7 months before diagnosis [3.7 work loss days in patients with RA *vs* 3.2 days in same-sex siblings, corresponding to an adjusted mean difference of 0.56 days (95%CI 0.07–1.05; Fig. 1)].

### Main analysis

In the first year after diagnosis, patients with RA had significantly more work loss compared with same-sex siblings, in both diagnosis periods (Fig. 1, Table 2). For patients diagnosed between 2006 and 2011, the adjusted mean difference in the first year was 47 days (95%CI 39–54) corresponding to 96% higher work loss relative to same-sex siblings, while for patients diagnosed between 2012 and 2020, the difference was 26 days (95%CI 20–32) corresponding to 67% higher work loss relative to same-sex siblings (P_interaction_ < 0.001 for early *vs* late diagnosis period).

**Table 2:** Adjusted annual work loss days and percentage differences between patients with rheumatoid arthritis and their same-sex siblings, overall and by subgroups.

Group	Follow-up from RA diagnosis	Mean RA	Mean same-sex siblings	Adjusted mean difference (95%CI)^a^	Percentage difference^b^
Overall					
	Year 0–1	78	44	35 (31–40)	80%
	Year >1–2	68	45	24 (19–29)	53%
	Years >2–10	69	47	24 (18–29)	51%
Diagnosed 2006–2011					
	Year 0–1	95	49	47 (39–54)	96%
	Year >1–2	80	47	34 (26–42)	72%
	Years >2–10	74	48	29 (22–36)	60%
Diagnosed 2012–2020					
	Year 0–1	64	39	26 (20–32)	67%
	Year >1–2	56	44	14 (7–21)	32%
	Years >2–10	57	46	11 (4–19)	24%
Men					
	Year 0–1	63	35	28 (20–37)	80%
	Year >1–2	55	37	18 (9–27)	49%
	Years >2–10	55	38	18 (9–27)	47%
Women					
	Year 0–1	84	47	38 (32–44)	81%
	Year >1–2	73	49	26 (20–32)	53%
	Years >2–10	75	51	26 (19–32)	51%
Age <50 years					
	Year 0–1	59	32	28 (22–34)	88%
	Year >1–2	51	34	18 (12–24)	53%
	Years >2–10	53	36	18 (12–25)	50%
Age ≥50 years					
	Year 0–1	95	54	43 (35–50)	80%
	Year >1–2	83	55	30 (22–38)	55%
	Years >2–10	84	57	27 (19–35)	47%
Low education (high school or less)					
	Year 0–1	98	53	46 (38–53)	87%
	Year >1–2	87	55	32 (25–40)	58%
	Years >2–10	84	56	30 (22–38)	54%
High education (some tertiary education)					
	Year 0–1	40	19	22 (14–29)	116%
	Year >1–2	33	20	14 (7–22)	70%
	Years >2–10	34	22	13 (5–20)	59%
Seropositive					
	Year 0–1	76	45	32 (26–38)	71%
	Year >1–2	67	48	20 (13–26)	42%
	Years >2–10	68	49	20 (13–26)	41%
Seronegative					
	Year 0–1	81	42	42 (33–50)	100%
	Year >1–2	71	41	32 (24–41)	78%
	Years >2–10	71	44	31 (22–39)	70%

a The estimate represents the adjusted mean annual difference in work loss days between RA patients and their same-sex siblings, obtained from a linear regression model that accounts for age, age squared, sex, year of observation and same-sex sibling fixed effects. Adjusted mean differences and 95% confidence intervals are rounded to the nearest day.

b Percentage difference is adjusted mean difference in work loss between patients with RA and same-sex siblings divided by the work loss of the same-sex siblings.

Work loss remained elevated, but less pronounced, from year 2 to year 10 following the RA diagnosis (Fig. 1, Table 2). For patients diagnosed between 2006 and 2011, the adjusted mean difference from year 2–10 was 29 days annually (95%CI 22–36), or 60% higher than same-sex siblings. For patients diagnosed between 2012 and 2020, the adjusted mean difference was 11 days annually (95%CI 4–19), or 24% higher than same-sex siblings (P_interaction_ = 0.001 for early *vs* late diagnosis period). Including comorbidities among the covariates had little to no effect on these results (Supplementary Table S2).

### Peak work loss

The largest difference in work loss between patients with RA and same-sex siblings occurred the month following diagnosis (Fig. 1). In patients diagnosed 2006–2011, work loss peaked in patients at 9.5 days per month compared with 4.2 days per month for same-sex siblings (adjusted mean difference 5.5, 95%CI 4.8–6.2). In patients diagnosed 2012–2020, work loss peaked in patients with RA at 6.6 days per month *vs* 3.2 for same-sex siblings (adjusted mean difference 3.5, 95%CI 2.9–4.0).

### Trends by 3-year intervals

**Figure 2:**
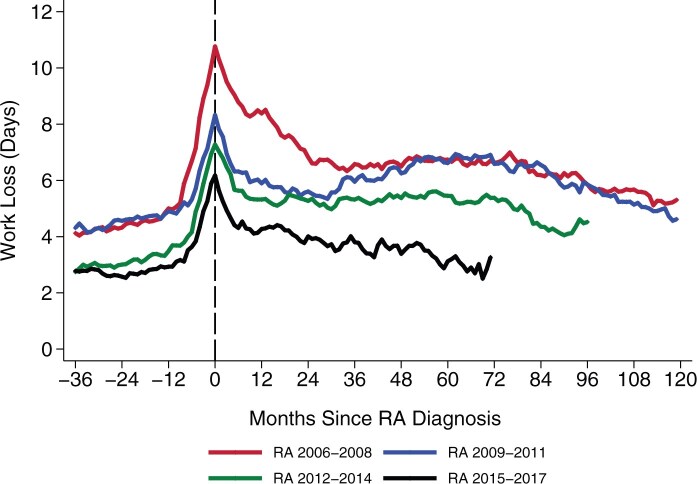
Monthly work loss for patients with rheumatoid arthritis (RA), stratified by diagnosis period, and measured from 36 months before to 120 months after RA diagnosis.

When stratified by 3-year intervals, work loss for patients with RA peaked during the month after diagnosis in all four cohorts (Fig. 2). The earliest 2006–2008 cohort exhibited the highest work loss, while more recent cohorts had progressively lower peak levels of work loss and faster subsequent declines. In the latest period (2015–2017) the point estimates for the monthly differences in work loss between patients with RA and same-sex siblings were small and statistically insignificant from 2 to 5 years after diagnosis (Supplementary Fig. S2).

### Work loss distribution

**Figure 3:**
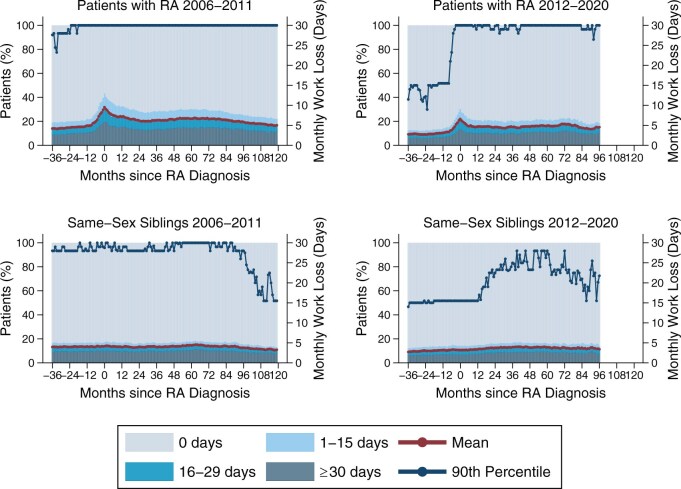
Monthly work loss for patients with rheumatoid arthritis (RA) (top panels) and same-sex siblings (bottom panels) stratified by diagnosis period (2006–2011 *vs* 2012–2020). Left axis displays distribution of monthly work loss days in four categories. Right axis displays mean and 90th percentile values for monthly work loss. All outcomes are measured from 36 months before to 120 months after RA diagnosis

The distribution of work loss was skewed in patients and same-sex siblings, with a small fraction of individuals generating most of the total work loss. At peak work loss 1 month after diagnosis, 20% of patients with RA diagnosed between 2006 and 2011 had full work loss (30 days per month) whereas the corresponding fraction was 14% among patients diagnosed between 2012 and 2020 (*P* < 0.001) (Fig. 3). Among same-sex siblings, the corresponding fractions for the early and late diagnosis periods were 9.6% and 6.7%, respectively.

The share of same-sex siblings with no registered work loss in the one year prior to the index date and the two following years was 59% in 2008 and rose to 63% in 2018. Among patients with RA, the share with no registered work loss was 26% in 2008 and rose to 41% in 2018 (Supplementary Fig. S3).

### Effect modification by sex, age, education level and seropositivity

No statistically significant effect modifications were found by sex or seropositivity during years 2–10 after diagnosis, but a larger increase in work loss relative to same-sex siblings for older (27 days, 95%CI 19–35) compared with younger patients (18 days, 95%CI 12–25; P_interaction_ = 0.023). There was also a larger increase in work loss for patients with RA relative to same-sex siblings within the group with low education (30 days, 95%CI 22–38) compared with the group with high education (13 days, 95%CI 5–20; P_interaction_ = 0.002).

## Discussion

We report that individuals diagnosed with RA have more work loss than their same-sex siblings, with peak work loss occurring shortly after diagnosis. Importantly, however, our results show that the trajectory of work loss has changed over diagnosis periods. An RA diagnosis between 2012 and 2020 was associated with lower peak work loss, faster improvement and a smaller difference in work loss *vs* same-sex siblings, than a diagnosis between 2006 and 2011. Furthermore, the time from initial rise in work loss among future patients (relative to same-sex siblings) until date of diagnosis was shorter in the 2012–2020 *vs* 2006–2011 diagnosis period. These findings underscore the evolving landscape of RA management and beneficial impact on workplace outcomes.

Although large sample sizes can make small differences statistically significant, the improvements in our study were substantial and driven by fewer patients experiencing full work loss. This reflects meaningful gains at the individual and societal level.

### Previous research

Our findings align with previous research showing that work loss in RA is skewed, with no clear sex differences but greater work loss among older individuals and those with lower education. To the best of our knowledge, this is the first same-sex sibling study on the association between RA and work loss, and the first study to investigate calendar period trends in longitudinal work loss using such a study design. Previous studies in Sweden have shown that RA significantly impacts work loss in the months surrounding diagnosis and over the long-term [12, 13]. These studies highlight substantial work absences due to RA but generally do not explore how advancements in treatment and other time trends might have influenced work loss. A register-based study from Finland found a decline in work loss from 2000 to 2008 in patients with RA, suggesting that improvements in disease management were already being translated into better work outcomes [17]. Further, a recent study from Germany using repeated cross-sections showed decreasing proportions of disability pension and lower absenteeism from 2010 to 2022 in patients with inflammatory rheumatic musculoskeletal diseases, including RA [22].

We recently reported similar findings for earnings, with a significant earnings decline in patients *vs* same-sex siblings, with the most pronounced decline in the years immediately after diagnosis, but with calendar year effects showing improved outcomes for patients with RA over time [16]. Earnings and work loss studies complement each other in understanding the impact of RA. While earnings studies focus on work productivity and loss of income, work loss studies provide valuable insights into the duration and extent of absences due to the disease. In Sweden, earnings data capture immediate financial impacts, including the first 14 days of work loss and longer-term absences, while work loss studies provide a broader perspective on overall absence patterns but do not include the first 14 days of sick leave spells.

### Strengths

This study has several strengths, including its population-based design and large sample size. Objective and complete outcome data on work loss ensure a high level of accuracy. Additionally, the same-sex sibling comparison controls for shared genetic and environmental factors, allowing for a more precise assessment of the impact of RA on work loss [23]. The long follow-up period made it possible to investigate calendar period trends during an era with increasing biologic and non-biologic treatment alternatives.

### Limitations

A limitation of this study is the exclusion of short sick leave episodes (<14 days), compensated by employers and not captured in our data. This may underestimate total work loss days, particularly for individuals with frequent short-term absences. However, we believe this has limited impact on the overall results, as work loss is heavily skewed and long-term absences account for the majority of total work loss.

An additional limitation is that not all patients have a same-sex sibling, which means that we cannot be certain about the generalizability of the findings to the whole population with RA including those with opposite sex- or no siblings. The same-sex sibling design remains, however, a robust methodological approach to account for potential confounding in a different way than comparisons with matched general population controls or standard multivariable adjustment. The sibling design strengthens causal inference by accounting for shared familial factors, but residual confounding cannot be excluded since RA status is not randomly assigned. These findings may not be generalizable to patients with RA in other countries due to differences in social insurance systems and treatment practices.

### Conclusion

Our study highlights that RA continues to be associated with higher work loss, particularly around the time of diagnosis, but with lower impact observed in more recent periods. While overall work loss decreased in both groups, the relative difference between patients and siblings also decreased over diagnosis periods, as did time from work loss onset to diagnosis, indicating that advances in RA care contributed beyond general improvements. These findings align with previous research on loss of earnings, showing the most pronounced impact shortly after diagnosis and improvements over time. Together, the results underscore how the socioeconomic burden of RA is evolving, and that earlier diagnosis, as well as improved treatment strategies, including access to more treatment alternatives, may improve workplace outcomes for patients with RA. These improvements not only reduce the societal burden of RA but also enhance the daily lives and work participation of patients.

## Supplementary Material

keag124_Supplementary_Data

## Data Availability

Data may be obtained from a third party and are not publicly available. The study data forms part of a register linkage performed by Karolinska Institutet, and for which further sharing of the data is limited by legal restrictions.
