# Supplementary material for: Incident rheumatoid arthritis and work loss: a nationwide sibling comparison study
Source: Rheumatology (Oxford). 2026 Mar 19;65(4):keag124. doi: 10.1093/rheumatology/keag124 (PMC13131211; doi:10.1093/rheumatology/keag124)
Supplement: keag124_Supplementary_Data [file keag124_supplementary_data.docx]

**Supplementary Appendix**

**Incident Rheumatoid Arthritis and Work Loss:
A Nationwide Sibling Comparison Study**

*Heather Miller^a^, Gustaf Bruze^a^, Kari Johansson^a^, Johan Askling^a,b^, Martin Neovius^a,c^*

*^a^*Clinical Epidemiology Division, Department of Medicine Solna,

Karolinska Institutet, Stockholm, Sweden

*^b^*Rheumatology, Theme Inflammation and Ageing,

Karolinska University Hospital, Stockholm, Sweden

^c^Department of Public Health Sciences, Thompson School of
Social Work & Public Health, University of Hawai‘i at Mānoa, USA

**Table of Contents**

| **Item** | **Description** | **Page** |
| --- | --- | --- |
| Table S1 | Diagnosis codes for the National Patient Register | 2 |
| Table S2 | Characteristics of participants | 3 |
| Table S3 | Sensitivity analysis including comorbidities among covariates | 4 |
| Figure S1 | Flow-chart | 5 |
| Figure S2 | Work loss of patients with RA and same-sex siblings by diagnosis year (3 yr intervals) | 6 |
| Figure S3 | Share of patients with RA and same-sex siblings with no compensated work loss | 7 |

**Supplement Table S1.** Baseline comorbidities and corresponding diagnosis codes from the National Patient Register.

| **Condition** | **ICD 10 Codes** |
| --- | --- |
| Rheumatoid Arthritis (RA) | M05, M06.0, M06.2, M06.3, M06.8, M06.9, M12.3 |
| Cardiovascular Disease | I00-I99 |
| Psychiatric Disorder | F00-F99 |
| Substance Use Disorder | F10-F19 |
| Musculoskeletal Disorder | M00-M99 |

**Supplement Table S2.** Baseline characteristics of patients with rheumatoid arthritis (RA) diagnosed between 2006 and 2020 and their same-sex siblings.

|  | Patients with RA (n=3850) | Same-Sex Siblings  (n=4422) |
| --- | --- | --- |
|  |  |  |
| Women, n (%) | 2719 (70.6) | 3113 (70.4) |
| Married, n (%) | 1843 (47.9) | 2081 (47.1) |
| Seropositive RA, n (%) | 2552 (66.3) | n.a. |
| Age (Years) at Diagnosis/Index Date |  |  |
| Mean (SD) | 48.2 (7.8) | 48.3 (7.6) |
| Median (p25-p75) | 49.8 (42.5-54.7) | 49.8 (42.7-54.6) |
| Year of Diagnosis, Median (p25-p75) | 2012 (2009-2016) | 2012 (2009-2016) |
| Education and Earnings |  |  |
| Primary School, n (%) | 527 (13.7) | 594 (13.4) |
| High School, n (%) | 2037 (52.9) | 2313 (52.3) |
| University, n (%) | 1283 (33.3) | 1506 (34.1) |
| *Education Missing, n (%)* | *3 (0.1)* | *9 (0.2)* |
| Earnings, Mean (SD) | 275,300 (204,700) | 292,900 (209,100) |
| Comorbidities* |  |  |
| Cardiovascular Disease, n (%) | 317 (8.2) | 360 (8.1) |
| Psychiatric Disorder, n (%) | 314 (8.2) | 366 (8.3) |
| Substance Use Disorder, n (%) | 85 (2.2) | 77 (1.7) |
| Musculoskeletal Disorder (Any), n (%) | 1215 (31.6) | 776 (17.5) |
| Musculoskeletal Disorder (Inpatient), n (%) | 175 (4.5) | 128 (2.9) |

*Retrieved from the National Patient Register, including inpatient and non-primary outpatient care, from 5 years before to 1 year before RA diagnosis.

**Supplement Table S3.** Adjusted annual work loss days and percentage differences between patients with rheumatoid arthritis and their same-sex siblings, overall and by subgroups.

| Group | | Follow-Up from RA Diagnosis | Mean  RA | Mean Same-Sex  Siblings | Adjusted Mean  Difference (95% CI) ^a^ | Percentage  Difference ^b^ |
| --- | --- | --- | --- | --- | --- | --- |
| Overall | |  |  |  |  |  |
|  | | Year 0-1 | 78 | 44 | 35 (31-40) | 80% |
|  | | Year >1-2 | 68 | 45 | 24 (19-29) | 53% |
|  | | Years >2-10 | 69 | 47 | 24 (19-29) | 51% |
| Diagnosed 2006-2011 | |  |  |  |  |  |
|  | | Year 0-1 | 95 | 49 | 47 (40-55) | 96% |
|  | | Year >1-2 | 80 | 47 | 35 (27-42) | 72% |
|  | | Years >2-10 | 74 | 48 | 29 (23-36) | 60% |
| Diagnosed 2012-2020 | |  |  |  |  |  |
|  | | Year 0-1 | 64 | 39 | 25 (20-31) | 67% |
|  | | Year >1-2 | 56 | 44 | 14 (7-20) | 32% |
|  | | Years >2-10 | 57 | 46 | 12 (5-19) | 24% |
| Men | |  |  |  |  |  |
|  | | Year 0-1 | 63 | 35 | 29 (21-37) | 80% |
|  | | Year >1-2 | 55 | 37 | 19 (10-28) | 49% |
|  | | Years >2-10 | 55 | 38 | 19 (10-28) | 47% |
| Women | |  |  |  |  |  |
|  | | Year 0-1 | 84 | 47 | 38 (32-44) | 81% |
|  | | Year >1-2 | 73 | 49 | 26 (20-32) | 53% |
|  | | Years >2-10 | 75 | 51 | 26 (20-32) | 51% |
| Age <50 years | |  |  |  |  |  |
|  | | Year 0-1 | 59 | 32 | 27 (22-33) | 88% |
|  | | Year >1-2 | 51 | 34 | 17 (11-23) | 53% |
|  | | Years >2-10 | 53 | 36 | 18 (12-25) | 50% |
| Age ≥50 years | |  |  |  |  |  |
|  | | Year 0-1 | 95 | 54 | 43 (36-50) | 80% |
|  | | Year >1-2 | 83 | 55 | 31 (23-38) | 55% |
|  | Years >2-10 | | 84 | 57 | 28 (20-35) | 47% |
| Low education  (High school or less) |  | |  |  |  |  |
|  | Year 0-1 | | 98 | 53 | 46 (39-53) | 87% |
|  | Year >1-2 | | 87 | 55 | 33 (26-41) | 58% |
|  | Years >2-10 | | 84 | 56 | 30 (23-38) | 54% |
| High education  (Some tertiary education) | | |  |  |  |  |
|  | Year 0-1 | | 40 | 19 | 21 (14-28) | 116% |
|  | Year >1-2 | | 33 | 20 | 13 (6-21) | 70% |
|  | Years >2-10 | | 34 | 22 | 13 (5-20) | 59% |
| Seropositive |  | |  |  |  |  |
|  | Year 0-1 | | 76 | 45 | 34 (28-40) | 76% |
|  | Year >1-2 | | 67 | 48 | 21 (15-27) | 44% |
|  | Years >2-10 | | 68 | 49 | 22 (15-28) | 45% |
| Seronegative |  | |  |  |  |  |
|  | Year 0-1 | | 81 | 42 | 39 (31-47) | 93% |
|  | Year >1-2 | | 71 | 41 | 29 (21-38) | 71% |
|  | Years >2-10 | | 71 | 44 | 28 (20-37) | 64% |

^a^ The estimate represents the adjusted mean annual difference in work loss days between RA patients and their same-sex siblings, obtained from a linear regression model that accounts for age, age squared, sex, year of observation, comorbidities (previous cardiovascular disease, psychiatric disorder, and substance use disorder) and same-sex sibling fixed effects. Adjusted mean differences and 95% confidence intervals are rounded to the nearest day.

^b^ Percentage difference is adjusted mean difference in work loss between patients with RA and same-sex siblings divided by the work loss of the same-sex siblings.


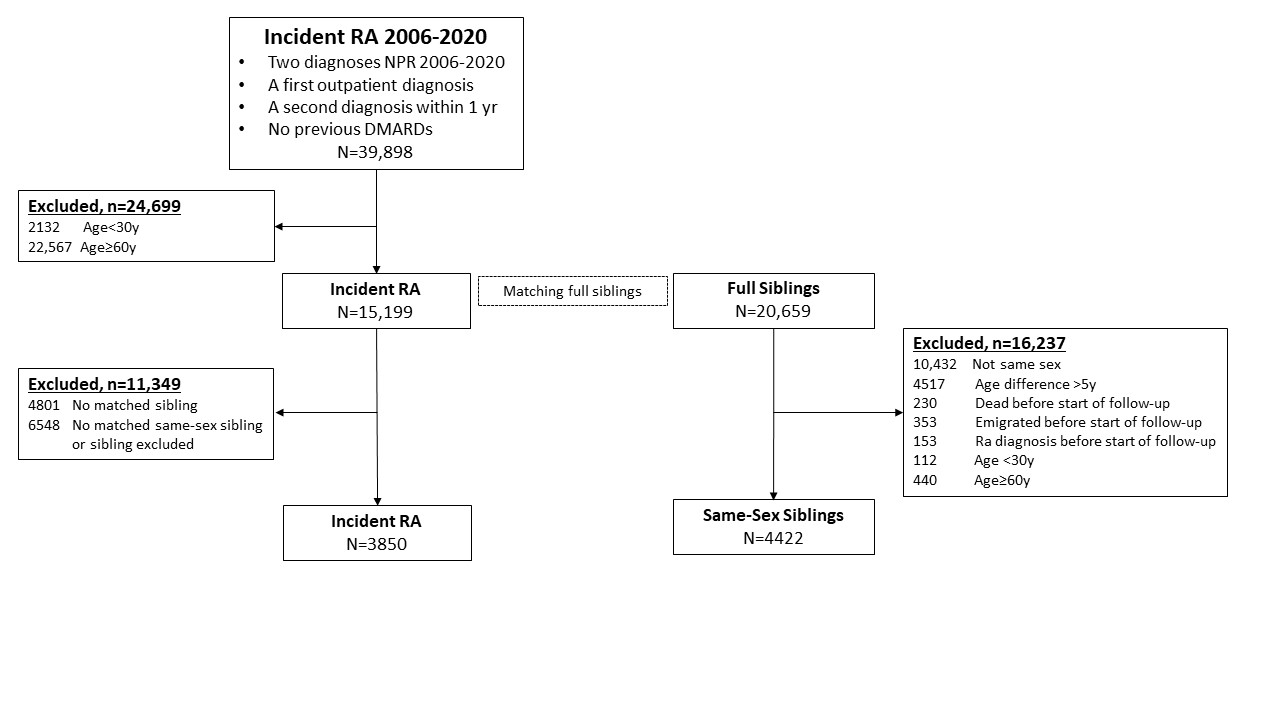


**Figure S1**. Flow-chart for selection of patients with RA and their same-sex siblings.

**Figure S2**. Monthly work loss for patients with RA and same-sex siblings by diagnosis period.

**Top panels:** Mean monthly work loss days for patients with rheumatoid arthritis (RA) and their same-sex siblings.
**Bottom panels:** Annual mean difference (with 95th percentiles) in work loss between patients and their same-sex siblings estimated using multiple regression adjusted for age, age², and calendar year, with sibling fixed effects. Standard errors were clustered at the sibling level.

**Figure S3**. Share of patients with RA and same-sex siblings (%) with no compensated work loss neither the 1 year before nor the 2 years after diagnosis,
by calendar year of diagnosis.
